# Atmospheric Moisture Variability and Transmission of Hemorrhagic Fever with Renal Syndrome in Changsha City, Mainland China, 1991–2010

**DOI:** 10.1371/journal.pntd.0002260

**Published:** 2013-06-06

**Authors:** Hong Xiao, Huai-Yu Tian, Bernard Cazelles, Xiu-Jun Li, Shi-Lu Tong, Li-Dong Gao, Jian-Xin Qin, Xiao-Ling Lin, Hai-Ning Liu, Xi-Xing Zhang

**Affiliations:** 1 College of Resources and Environment Science, Hunan Normal University, Changsha, China; 2 Ecologie & Evolution, UMR 7625, UPMC-ENS, Paris, France; 3 UMMISCO UMI 209 IRD - UPMC, Bondy, France; 4 School of Public Health, Shandong University, Jinan, China; 5 School of Public Health and Institute of Health and Biomedical Innovation, Queensland University of Technology, Brisbane, Queensland, Australia; 6 Hunan Provincial Center for Disease Control and Prevention, Changsha, China; 7 Changsha Municipal Center for Disease Control and Prevention, Changsha , China; Centers for Disease Control and Prevention, United States of America

## Abstract

**Background:**

The transmission of hemorrhagic fever with renal syndrome (HFRS) is influenced by environmental determinants. This study aimed to explore the association between atmospheric moisture variability and the transmission of hemorrhagic fever with renal syndrome (HFRS) for the period of 1991–2010 in Changsha, China.

**Methods and Findings:**

Wavelet analyses were performed by using monthly reported time series data of HFRS cases to detect and quantify the periodicity of HFRS. A generalized linear model with a Poisson distribution and a log link model were used to quantify the relationship between climate and HFRS cases, highlighting the importance of moisture conditions. There was a continuous annual oscillation mode and multi-annual cycle around 3–4 years from 1994 to 1999. There was a significant association of HFRS incidence with moisture conditions and the Multivariate El Niño–Southern Oscillation Index (MEI). Particularly, atmospheric moisture has a significant effect on the propagation of HFRS; annual incidence of HFRS was positively correlated with annual precipitation and annual mean absolute humidity.

**Conclusions:**

The final model had good accuracy in forecasting the occurrence of HFRS and moisture condition can be used in disease surveillance and risk management to provide early warning of potential epidemics of this disease.

## Author Summary

Hemorrhagic fever with renal syndrome (HFRS), a rodentborne disease caused by Hantaviruses, is characterized by fever, haemorrhage, headache, back pain, abdominal pain, and acute kidney injury. At present, it is endemic in all 31 provinces, autonomous regions, and metropolitan areas in mainland China where human cases account for 90% of the total global cases. Infection rates and population dynamics of hosts are thought to be influenced by climatic factors, especially humidity. Some studies have found that hantaviruses are limited in their spread to high-humidity environments for extended ex vivo stability. Here we provide the evidence that HFRS incidence was strongly associated with moisture conditions, including seasonal variation and annual situation, in Changsha, mainland China, 1991–2010. The results most likely indicate that moisture not only influences growth of food sources that determine rodent population size, thereby affecting the HFRS transmission, but also directly influences rodent activity and hantavirus infectivity. These findings offer insights in understanding possible causes of HFRS transmission, and can be used in disease surveillance and risk management to provide early warning of potential epidemics of this disease.

## Introduction

Hemorrhagic fever with renal syndrome (HFRS) is a rodent-borne disease caused by hantavirus, and is characterized by fever, hemorrhage, kidney damage, and hypotension. HFRS has been recognized as an important public health problem in Hunan Province, which is one of the most seriously affected areas in mainland China [1], [2]. HFRS was first detected in Hunan Province in 1963, and the highest incidence of the disease was recorded at 13.33/100,000 in 1985. Changsha, the capital city of Hunan Province, also has a high prevalence of HFRS. Among Changsha areas, the highest incidence of HFRS was recorded in Ningxiang County, at 101.68/100, 000 in 1994.

Many studies showed that the incidence rates of HFRS and the population dynamics of hosts are influenced by climatic factors, especially humidity. In Belgium, high soil moisture was significantly associated with the number of nephropathia epidemica (NE) cases [3]. In northeast China, relative humidity was positively associated with transmission of HFRS [4]. The highest HFRS incidence was recorded at 6.4/100,000, in semi-humid areas with precipitation greater than 400 mm. In China, nearly 50% of HFRS cases have been in areas with precipitation greater than 800 mm [2]. Furthermore, under laboratory conditions, high humidity been proven to be beneficial for virus survival in the *ex vivo* environment [5]. Some studies also found that hantaviruses are limited in their spread to high-humidity environments for extended *ex vivo* stability [6]. However, little is known how the moisture condition, including seasonal variation and annual situation, influence the HFRS transmission in a relatively long period of time.

In this study, we used wavelet analysis to investigate variations in dominant periodic cycles across the time series of HFRS, for quantifying the relationship between moisture condition and HFRS in the long run. We also aimed to develop a moisture condition-based forecasting model for the control and prevention of HFRS.

## Methods

### Study area

**Figure 1:**
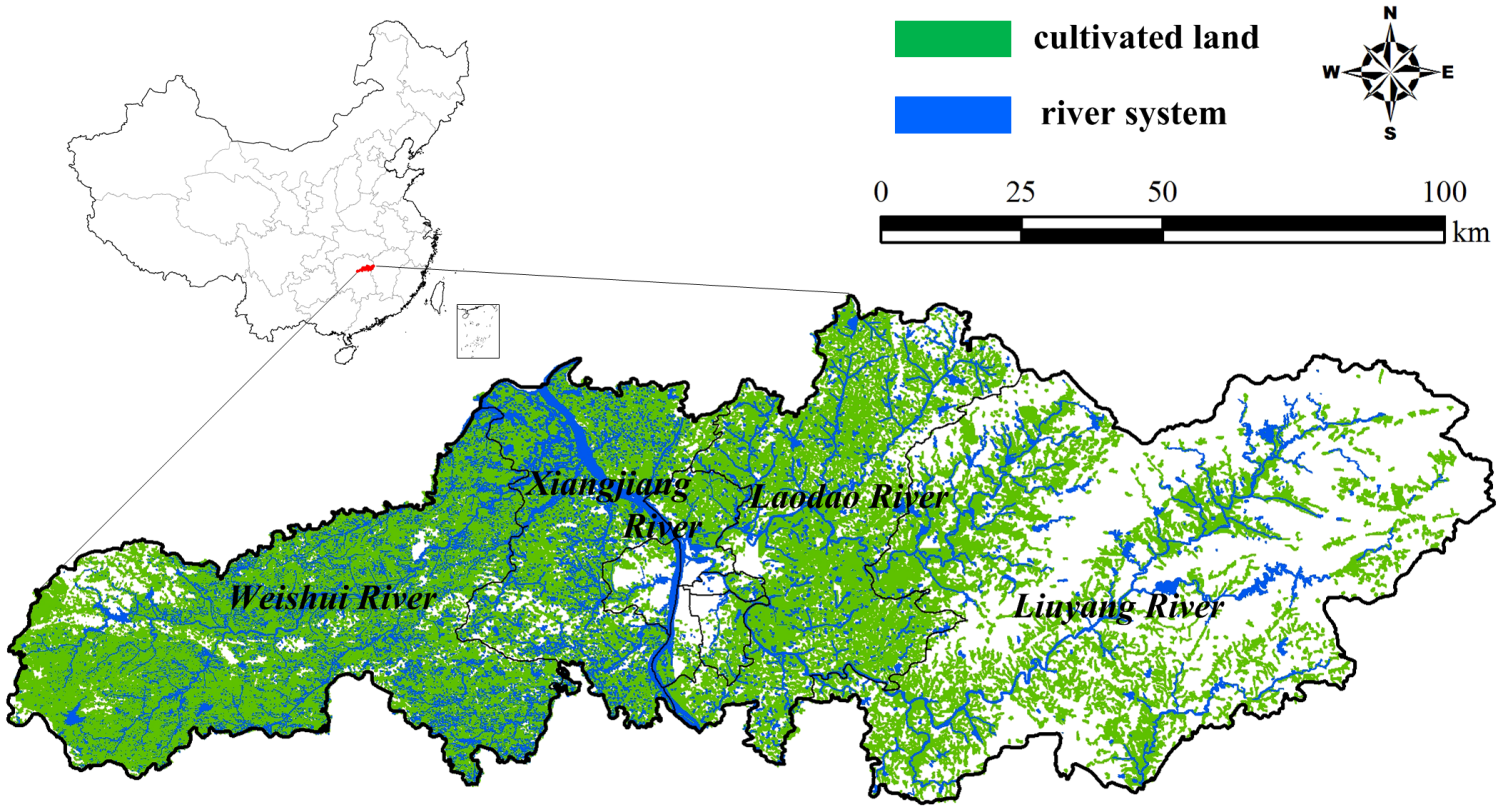
Geographic location of Changsha, China.

Changsha is a city with a total land area of 118,000 square kilometers and a population of about seven million in 2010. Changsha comprises the Xiangjiang River alluvial plain, part of the Central Plain of China. The Xiangjiang, Weishui, Laodao, and Liuyang rivers and their branches flow through the city. These have promoted development of the region's traditional agricultural economy, with subtropical double-harvest rice cultivation of major importance. These environmental conditions provide the opportunity for HFRS to persist and spread (Figure 1).

### Ethical review

The present study was reviewed by the research institutional review board of Hunan CDC, and it is found that utilization of disease surveillance data did not require oversight by an ethics committee. The data were analyzed anonymously, using publicly available secondary data, therefore no ethics statement is required for this work.

### HFRS and climatic data

Records of HFRS cases from 1991 to 2010 were obtained from the the Hunan Notifiable Disease Surveillance System (HNDSS). The data were from a passive surveillance system and all HFRS cases were first diagnosed by clinical symptoms, as defined by a national standard [7]. Clinical diagnosis criteria include: A person who had exposure to rodents and their feces, saliva and urine urine or who had traveled to the HFRS endemic area within two months prior to the onset of illness, and who had an acute illness with at least two of the following clinical symptoms: fever, chill, hemorrhage, headache, back pain, abdominal pain, acute renal dysfunction, and hypotension. Then, antibody-based serological tests were used (e.g. MacELISA, IFA). A confirmed case of HFRS had to have had at least one of the laboratory criteria for diagnosis. Cumulative cases for each month over the study period were calculated to reflect seasonal fluctuations. In addition, the surveillance strategies for HFRS in Changsha have not changed during the study period.

Monthly climatic data from 1991 to 2010 in Changsha were collected from the China Meteorological Data Sharing Service System (http://cdc.cma.gov.cn/index.jsp). Climatic factors included monthly mean relative humidity and monthly accumulated precipitation. Absolute humidity was generated from a transformation of air pressure, relative humidity and temperature:
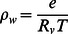
(1)

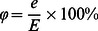
(2)

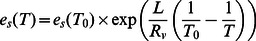
(3)where *ρ*_w_ is absolute humidity, *e* is vapor pressure, *Rv* is the gas constant of water evaporation, *L* is the latent heat of water evaporation, *e_s_*(*T*) is the saturation vapor pressure at the temperature *T*, *φ* is relative humidity and *E* is saturation vapor pressure.

The multivariate El Niño–Southern Oscillation index was available from the Earth System Research Laboratory of the National Oceanic and Atmospheric Administration [8]. The index was used as an indicator of the global climate pattern on the six main observed variables over the tropical Pacific. These six variables are: sea-level pressure, zonal and meridional components of the surface wind, sea surface temperature, surface air temperature, and total cloudiness fraction of the sky.

### Wavelet approach

Wavelet analysis is suitable for investigating time series data from non-stationary systems [9], [10]. Wavelet analyses have been increasingly used to analyze various human infectious disease dynamics such as malaria [11], measles [12], influenza [13], leishmaniasis [14] and dengue [15], [16]. We conducted wavelet analysis on a time series of reported HFRS cases to detect and to quantify variability of HFRS incidence over time.

Wavelet analysis allows investigation and quantification of the temporal evolution of HFRS with different rhythmic components [9]. In this study, monthly HFRS incidences were square root transformed and normalized, and the trend was suppressed before analyses [9].

Bootstrap methods were used to quantify the statistical significance of the computed patterns [9]. As white noise or red noise hypothesis are not well-adapted to biological time series, we have chosen to use the synthetic series proposed by Rouyer et al. [17], called “beta-surrogates”, which display a similar autocorrelation structure as the original time series. Using this approach, we thus obtain surrogates that mimic the shape of the original time series by displaying a power spectrum with the same slope in the log scale, but without exactly reproducing it [17]. This allows to test if the variability of the observed time series or the association between two time series is no different to that expected from a random process that has similar properties than the process underlying the observed time series. Wavelet time series analyses were applied using well-established algorithms [18] implemented in MATLAB software (MathWorks, Inc.).

### Time-series adjusted Poisson regression

A generalized linear model (GLM) with a Poisson distribution and a log link [19] was performed after adjustment for autocorrelation, seasonality, and lag effects. Potential seasonal variation was controlled by including the dummy variable *month*. To control the long-term trends, we created indicator variables for “year” of onset in the model. The variable “Moisture Condition” reflects the annual atmospheric moisture variability including annual precipitation and annual mean absolute humidity. The core model was:
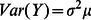
(4)where *Var* is the variance of the response and related to the mean μ, *σ* ^2^ is the dispersion parameter. Our model for counts in the presence of overdispersion can be written as:
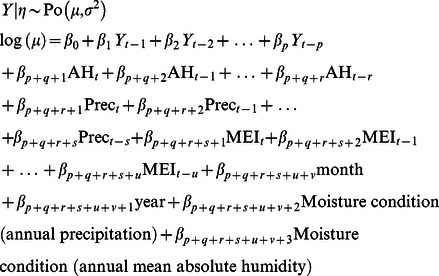
(5)


where p, q, r, s, u are lags determined by correlation analysis. *η* is the fitted model and Po is the Poisson distribution. Prec is the monthly accumulated precipitation and AH is the absolute humidity. MEI is the multivariate El Niño–Southern Oscillation index. Moisture Condition (annual precipitation) is calculated as sum of monthly precipitation for twelve months of the year and Moisture Condition (annual mean absolute humidity) is calculated by adding up all the monthly absolute humidity for twelve months of the year and then divide the sum by the number of months. A stepwise method was used to include variables, as long as there was significant improvement determined by calculation of the maximum likelihood ratio. GLM was fitted by using the R software package.

A cross-correlation analysis was used to detect of the association between climate factors and HFRS transmission, with a different time lags. The two time series were filtered to convert to white noise before computing the cross-correlation.

## Results

### Characteristics of HFRS epidemics

A total of 9,130 cases were confirmed in Changsha between 1991 and 2010, with a statistically significant gender difference. Individuals from lower socioeconomic groups (students, workers and peasants) comprised 93% of all HFRS cases, indicating these as high risk populations. The highest cumulative of annual incidence of HFRS during this period was 34.31/100,000 in 1997, and the lowest 0.73/100,000 in 2008.

### Frequency of HFRS epidemics and climate variability

**Figure 2:**
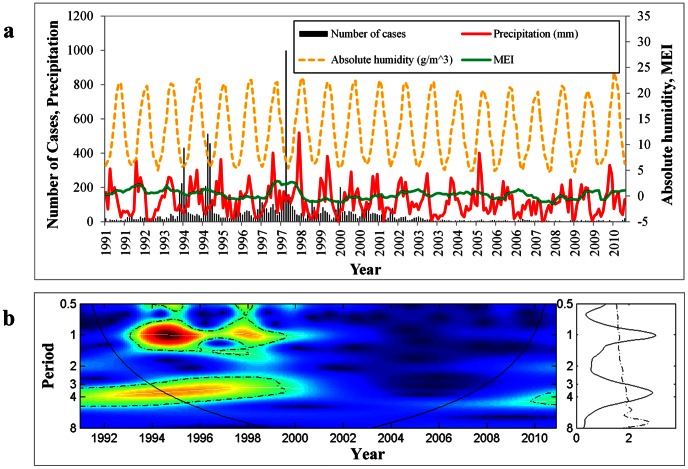
Wavelet power spectrum of HFRS incidence in Changsha. (*A*) Temporal variation in climatic variables and the number of hemorrhagic fever with renal syndrome (HFRS) cases in Changsha, 1991–2010. (*B*) The wavelet power spectrum of monthly number of HFRS cases by date of symptoms onset reported through the surveillance system in Changsha during the period 1991–2010 (square root transformed). The left panel illustrates the wavelet power spectrum for the different series (x-axia: time in year; y-axis: period in year). The power is coded from low values, in dark blue, to high values, in dark red. Statistically significant areas (threshold of 5% confidence interval) in wavelet power spectrum (left panels) are highlighted with dashed line; the cone of influence (region not influenced by edge effects) is also indicated. Finally, the right panels show the mean spectrm (solid line) with its significant threshold value of 5% (dashed line).

Figure 2 shows that HFRS cases in Changsha exhibit variability around two main temporal scales, with periods of 1 and 3–4 years. Both cyclic component were most pronounced in the 1990s. Timing of the breakpoint corresponds with apparent changes in both size of epidemics and patterns of seasonal variability, with appearance of the 1-year cycle in the wavelet spectrum. The wavelet spectrum also suggests that a 3–4-year period may have already been present prior to the 1990s. Clearly, there is a mark change in variability from the end of the 1990s to the beginning of the 2000s, accompanying a decline of overall incidence (Figure 2).

### Moisture condition and HFRS incidence

**Figure 3:**
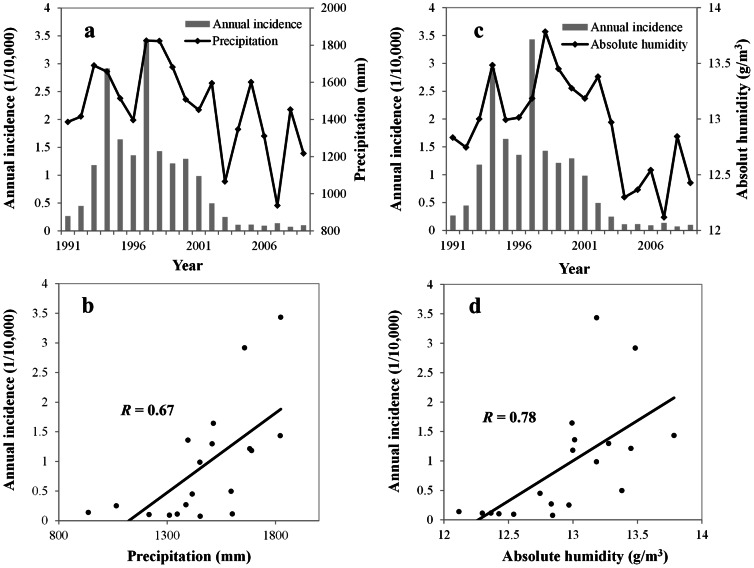
Annual HFRS cases and annual moisture condition, 1991–2010. (*A*) Temporal dynamics of annual precipitation and HFRS cases. (*B*) Scatterplot of annual precipitation and HFRS cases. (*C*) Temporal dynamics of annual mean AH and HFRS cases. (*D*) Scatterplot of annual mean AH and HFRS cases. The thick solid straight lines are linear regressions of annual HFRS cases and moisture condition.

Figure 3 shows that with increased annual precipitation, the annual incidence of HFRS cases began to increase from 1991 to 1995. From 1996 to 1997, annual precipitation increased quickly and reached a peak; the highest incidence of HFRS was recorded during the same period. With decreased annual precipitation, disease incidence began to decline. For example, annual precipitation began to decrease after 1998, and annual incidence of HFRS also dropped sharply.

In addition, the results reveal strong associations between annual HFRS incidence and annual precipitation (Spearman rho = 0.667, *P*<0.001), and between annual incidence of HFRS and annual mean AH (Spearman rho = 0.775, *P*<0.001). Disease incidence was associated with moisture over the long term.

### Predictive model based on moisture condition

As shown in Table 1, monthly HFRS cases are significantly correlated with precipitation, with the highest correlation coefficients having a lag of 5 months. The numbers of cases is correlated with AH and MEI, with a lag of 5 and 6 months, respectively..

**Table 1:** Maximum cross-correlation coefficients of monthly environmental variables and notifications of HFRS: Changsha, China, 1991–2010

Variable	Maximum coefficient^a^	Lag values (month)
Precipitation	0.482*	5
AH	0.41*	5
MEI	0.234*	6

* *p*<0.01.

a Chi-square test for cross-correlations.

Table 2 shows that the number of cases was third-order autoregressive, indicating that the number of cases in the current month was related to the number of cases in the previous 1, 2 and 3 months. HFRS cases had association with AH, precipitation and MEI. Seasonal and long-term trends also contributed to the number of cases. The final model suggests the following: A 1 g/m^3^ increase in AH was associated with a 47.2% (95% CI, 42.3–52.3%) increase in HFRS cases; a 1 mm increase in precipitation was associated with a 0.2% (95% CI, 0.1–0.3%) increase in HFRS cases; a 1 mm increase in the moisture condition (annual accumulated precipitation) was associated with a 0.1% (95% CI, 0.1–0.2%) increase in HFRS cases; a 1 g/m^3^ increase in the moisture condition (annual mean AH) was associated with a 2.6% (95% CI, 1.7–3.7%) increase in HFRS cases; and a 1-unit MEI rise was associated with a 64.5% (95% CI, 43.5–88.5%) increase in HFRS cases (Table 2). Only final parameter estimates of regression are presented in Table 2. Finally, we compared the final model to a model with the autocorrelation, year, and month components alone, results of AIC and pseudo-R^2^ were shown in Table S1.

**Table 2:** Summary of the model obtained for Changsha.^a^

Variable	IRR	95% CI	*P*
No. of cases, 3-month lag	1.003	1.002—1.003	<0.001
AH (g/m^3^), 5-month lag	1.472	1.423—1.523	<0.001
Precipitation (mm), 5-month lag	1.002	1.001—1.003	<0.001
MEI, 6-month lag	1.645	1.435—1.885	<0.001
Year of onset	0.910	0.904—0.916	<0.001
Moisture condition (Annual precipitation)	1.001	1.001—1.002	<0.001
Moisture condition (Annual mean absolute humidity)	1.026	1.017—1.037	<0.001

IRR, incidence rate ratio.

a Dummy variables for month were included in the final model.

**Figure 4:**
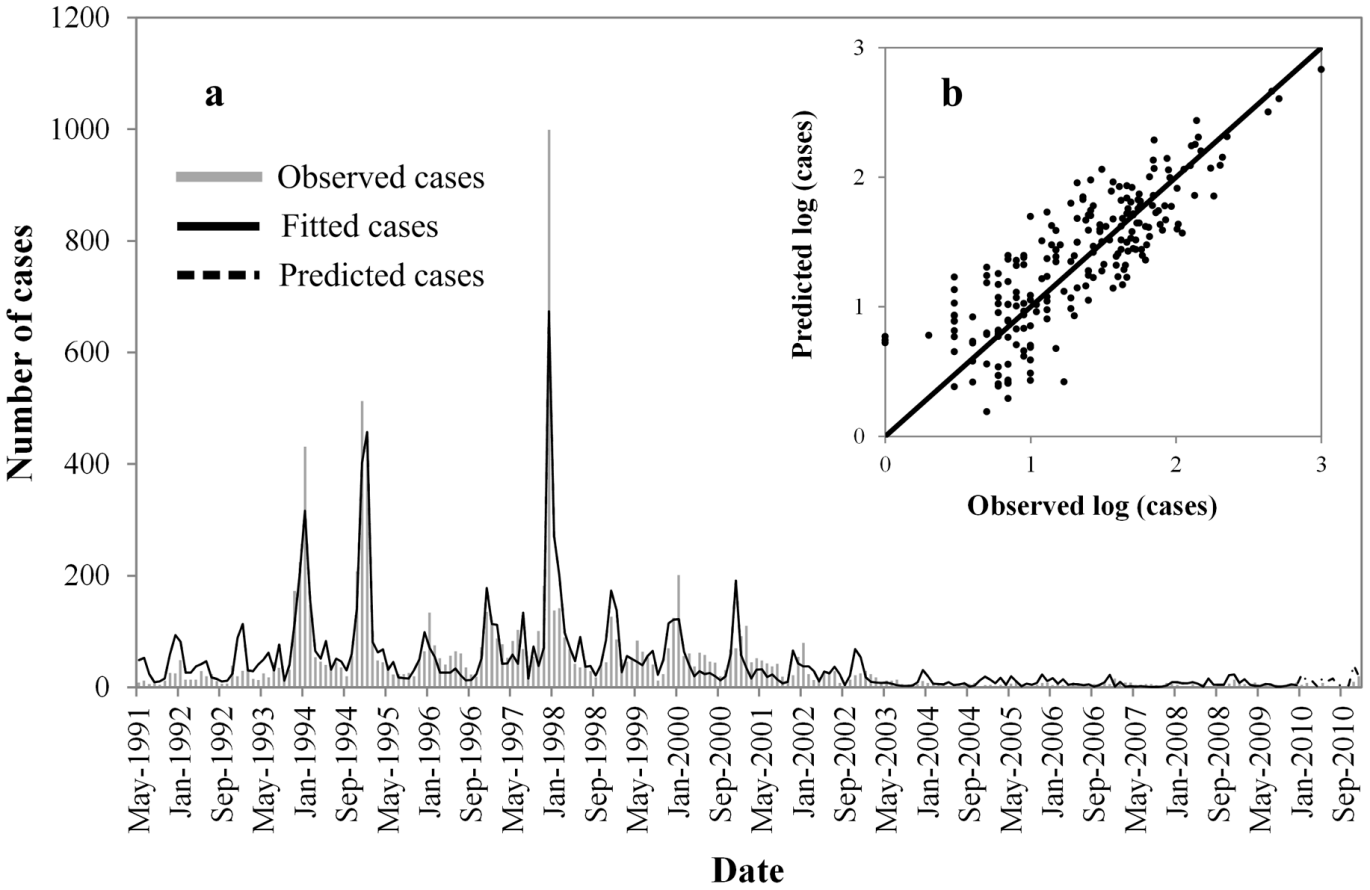
Observed versus predicted HFRS cases in Changsha. (*A*) Temporal dynamics, and (*B*) scatterplot.

As shown in Figure 4, the expected number of cases from the final model fitted very well with the observed number of cases in Changsha over the period 1991–2010, including peak values; the pseudo-R^2^ value for the fitted model was 83.64%. In diagnosis of the residuals of the model, a random distribution was observed, with no autocorrelation among them.

## Discussion

Considering the dynamics of HFRs in Changsha, there was a large decrease of HFRS incidence after 1998–1999. Possible explanations for this decrease could be due to the change in moisture conditions. We found a unique relationship between the moisture conditions and transmission of HFRS over a long period. The results most likely indicate that moisture not only influences growth of food sources that determine rodent population size, thereby affecting the HFRS transmission [20], [21], [22], but also directly influences rodent activity and hantavirus infectivity [6], [23]. The moisture condition (annual precipitation and annual mean AH) began to decrease after 1998, and the disease incidence also began to decline during the same period.

In our analyses, humidity and precipitation explained most the variance of the disease incidence. We postulate that because cultivated land and rivers cover most of the surface area in Changsha, these two landscape types can influence HFRS transmission by affecting the moisture conditions. Additionally, Changsha has been a traditional agricultural region where subtropical double-harvest rice has been historically cultivated, with at least 40% of the population as farmers or peasants. These combined factors support the transmission of HFRS.

A key finding of this study was that atmospheric moisture conditions are an important predictor of the intensity of HFRS transmission in central China, the annual moisture condition can be a good indicator for predicting HFRS epidemic in the long run. We found a consistent association of the HFRS transmission with monthly precipitation, AH with 5-month lags, annual accumulated precipitation and annual mean AH. Based on moisture conditions over the long term, we improved the performance of the final model, with pseudo-R^2^ value of 83.64%. In the model we used AH rather than RH because it affects the abundance and distribution of rodents more directly, since AH is the actual water vapor content of air irrespective of temperature [24].

The MEI seems to precede oscillations of HFRS in Changsha. This may reflect the timing of relationships between El Niño and local climate. El Niño can affect large-scale phenomena, which generate local climatic phenomena and thereby influence oscillations of epidemics. The El Niño effect may act as a pacemaker to affect local climatic moisture conditions, which in turn affect HFRS incidence.

Higher precipitation levels can induce severe HFRS epidemics in Changsha; however, excessive precipitation may cause flooding and a reduction of rodent population. The complexity of the links between HFRS dynamics and moisture is emphasized by the significant correlations in the annual mode over a long term observed in this study. In summer 1998, there was a large-scale weather anomaly in China, and the entire Yangtze River basin (including Changsha in the Xiangjiang River basin) suffered a severe flood event, the largest during the 20^th^ century apart from the 1954 floods. We speculate that a reduction in transmission was caused by declining rodent populations, reducing their contact with humans. As Thibault and Brown showed, an all-time record low number of rodents were captured the month following the downpour and resulting flooding [25].

The limitations of this study should also be acknowledged. Many factors can contribute to the transmission of HFRS. In addition to changes in climate conditions, the obvious decrease in the 2000s could also be due to other factors, e.g. changes in socio-economic status, human activities and movement, population immunity and/or local and national intervention programmes.

The findings here show that atmospheric moisture is an important predictor of HFRS incidence. This work may contribute to characterizing the temporal dynamics of HFRS in China, or in other countries with similar regional climate conditions. It may also have significant implications for integrating climate monitoring and disease surveillance data to effectively control and prevent the epidemics of HFRS.

## Supporting Information

Table S1 Summary of model performances.
(DOCX)
