# Supplementary material for: Atmospheric Moisture Variability and Transmission of Hemorrhagic Fever with Renal Syndrome in Changsha City, Mainland China, 1991–2010
Source: PLoS Negl Trop Dis. 2013 Jun 6;7(6):e2260. doi: 10.1371/journal.pntd.0002260 (PMC3674989; doi:10.1371/journal.pntd.0002260)
Supplement: Table S1 — Summary of model performances. (DOCX) [file pntd.0002260.s001.docx]

**Table S1** Summary of model performances

| Model | pseudo-R^2^ | AIC |
| --- | --- | --- |
| Final model | 0.836 | 4358 |
| Moisture variable only | 0.668 | 6058 |
| Autocorrelation variable only | 0.223 | 14134 |
| Year and month variable only | 0.425 | 10558 |
| Final model without year and month variable | 0.704 | 5408 |
| Final model without autocorrelation variable | 0.734 | 4880 |
| Final model without moisture variable | 0.565 | 7930 |
